# Machine learning approaches to predict age from accelerometer records of physical activity at biobank scale

**DOI:** 10.1371/journal.pdig.0000176

**Published:** 2023-01-24

**Authors:** Alan Le Goallec, Sasha Collin, M’Hamed Jabri, Samuel Diai, Théo Vincent, Chirag J. Patel

**Affiliations:** 1 Department of Biomedical Informatics, Harvard Medical School, Boston, Massachusetts, United States of America; 2 Department of Systems, Synthetic and Quantitative Biology, Harvard University, Cambridge, Massachusetts, United States of America; McGill University, CANADA

## Abstract

Physical activity improves quality of life and protects against age-related diseases. With age, physical activity tends to decrease, increasing vulnerability to disease in the elderly. In the following, we trained a neural network to predict age from 115,456 one week-long 100Hz wrist accelerometer recordings from the UK Biobank (mean absolute error = 3.7±0.2 years), using a variety of data structures to capture the complexity of real-world activity. We achieved this performance by preprocessing the raw frequency data as 2,271 scalar features, 113 time series, and four images. We defined accelerated aging for a participant as being predicted older than one’s actual age and identified both genetic and environmental exposure factors associated with the new phenotype. We performed a genome wide association on the accelerated aging phenotypes to estimate its heritability (h_g^2^ = 12.3±0.9%) and identified ten single nucleotide polymorphisms in close proximity to genes in a histone and olfactory cluster on chromosome six (e.g *HIST1H1C*, *OR5V1*). Similarly, we identified biomarkers (e.g blood pressure), clinical phenotypes (e.g chest pain), diseases (e.g hypertension), environmental (e.g smoking), and socioeconomic (e.g income and education) variables associated with accelerated aging. Physical activity-derived biological age is a complex phenotype associated with both genetic and non-genetic factors.

## Author summary

Physical activity improves quality of life and is also an important protective factor for prevalent age-related diseases and outcomes, such as diabetes and mortality. With age, physical activity tends to decrease, increasing vulnerability to disease in the elderly. Does physical activity measured from digital health devices predict one’s *biological age*? Biological age, as contrast to chronological age (the time that has elapsed since birth), is an indicator of the biological changes that accrue through time that are hypothesized to be one causal factor for age-related diseases. In the following, we trained machine learning models to predict age from 115,456 one week-long wrist accelerometer recordings from participants of the UK Biobank. We then found genetic, environmental, and behavioral factors associated with *accelerated age*, the difference between biological and chronological age, adding to the evidence of the biological plausibility of our new predictor. If reversable, summarizing complex physical activity into a biological age predictor may be a way of observing the effect of preventative efforts in real-time.

## Introduction

Physical activity improves quality of life and is associated with a decreased risk for age-related diseases such as cardiovascular diseases, diabetes, cancer and mental health [1] and reduces risk for mortality [2]. Furthermore, physical activity tends to decrease by 40–80% with age [3], making the elderly more vulnerable to the aforementioned diseases. Second, and relatedly, the manner, or gait [4], or the way humans are active (or inactive) changes with age. New sensor-based technologies, such as accelerometers, are poised to enable researchers to measure the type and gait of individuals’ physical activity.

With the aging of the world’s population, there is a need to define biological age, and identify the role of biological age in health and disease. According to Baker and Sprott, predictors of biological age are phenotypes that together better predict health-related outcomes and physiological function better than chronological age itself [5]. As reviewed extensively by Jhlhava and colleagues [6], there have been tremendous efforts in identifying biomarkers of aging, including telomeres, epigenetic, and other ‘omic based measures to estimate biological age. In contrast to chronological age–the time since an individual’s birth—biological age represents the state and condition of the individual’s body and is one underlying cause of age-related diseases. However, biological age must be estimated as a function of other age-related phenomena, such as life span or chronological age. For example, Horvath described [7] development of an algorithm whose inputs are thousands of epigenetic measurements along the genome that are trained on, or predict, chronological age. The prediction is defined as “biological age”.

We hypothesize that new sensors such as accelerometers that measure the level of physical activity of individuals throughout the day can also be used to predict age and biological age. Here, taking inspiration from Horvath and others, we define the biological age of a participant as the prediction outputted by the model and “accelerated aging” when biological age is greater than chronological age.

Others have predicted age using accelerometer data, such as ActiGraphs, to predict age [8,9]. For example, Rahman and colleagues analyzed participant health data from the National Health and Nutrition Examination Survey (NHANES) using deep learning approaches to predict the age of 14,631 individuals between the ages of 18–85 years of age. Physical activity “intensity”, or count, was input into these models [9]. Furthermore, several research teams have analyzed changes in gait to predict chronological age [10–18]. There have been efforts to predict components of “physical fitness”, such as cardiorespiratory health and its association with mortality [19]. Finally, use of newer tools, such as accelerometers, have been proposed using alternative measures of aging, such as time to death. For example, Strain et. al and Zhou et al built a survival models to predict mortality based on participant who were wearing wrist accelerometer data [20,21]. Specifically, the investigators processed the accelerometer data to derive energy expenditure and intensity of energy expenditure by estimating the type of physical activity (e.g., moderate to vigorous). They reported that higher energy expenditure was associated with a 20% lower risk for death for individuals that expended 20 versus 15 kilojoules per day, after 3.5 years of followup.

In the following, we leveraged 115,456 one week-long wrist accelerometer records collected from 103,680 participants aged 45–81 years to shed light on the complex relationship between aging and physical activity. We preprocessed the raw data into scalars, time series, and “images” that we then used as predictors to train deep neural network architectures to predict age. Next, we sought to understand the genetic and biological correlates of physical activity-based aging. We performed a genome wide association study [GWAS] to identify single nucleotide polymorphisms [SNPs] associated with physical-activity-defined biological or accelerated aging and estimated the total genetic variance. Similarly, we identified biomarkers, clinical phenotypes, diseases, family history, environmental and socioeconomic variables associated with physical activity-based accelerated aging.

## Methods

### Ethics statement

The Harvard internal review board (IRB) deemed the research as non-human subjects research (IRB: IRB16-2145). Formal consent was obtained by the UK Biobank (https://biobank.ctsu.ox.ac.uk/ukb/ukb/docs/Consent.pdf).

### Software availability

Our code can be found at https://github.com/Deep-Learning-and-Aging. For the genetics analysis, we used the BOLT-LMM [22,23] and BOLT-REML [24] software.

### Cohort Dataset: Participants of the UK Biobank

We leveraged the UK Biobank [25] cohort (project ID: 52887). The UKB cohort consists of data originating from a large biobank collected from 502,211 de-identified participants in the United Kingdom that were aged between 37 years and 74 years at enrollment (starting in 2006). Of these individuals, 103,688 UKB participants received wrist accelerometers that they wore for a week to measure their physical activity levels [26]. The accelerometer deployed in the UK Biobank is a device called the “Axivity AX3”, a wrist-worn 3-axis-based accelerometer. For more details about the device, we refer to Doherty and colleagues [26]. and the UK Biobank website (https://biobank.ndph.ox.ac.uk/showcase/refer.cgi?tk=o1TVIvRHH3O6rt8PrAUIDKsDmqcMs5NF259404&id=169649). See S1 Text on details on Hardware, Software, and design of training, tuning, and estimating prediction accuracy.

This sub-cohort was between 43.5 and 79 years of age (S1 Fig). Some of them repeated the experience during up to four “seasonal repeats” to evaluate the variability of their physical activity pattern between seasons, so the total number of accelerometer records amounts to 115,464. The raw data is available and consists of three leads (x, y and z axes) and a sampling frequency of 100 Hz with a dynamic range of ±8g (field 90001). A preprocessed version of the data is also available and consists of one lead that reports the average acceleration every five seconds, after filtering for noise and correcting for gravity (field 90004). For the preprocessed version, only one sample per participant is available, for a total of 103,687 samples.

### Defining biological age: predicted chronological age

We define biological age as the predicted chronological age. “Accelerated age” is when the predicted (or biological age) age is greater than chronological age and “decelerated age” is when the predicted age is less than the chronological age.

### Definition of the different physical activity dimensions

**Fig 1:**
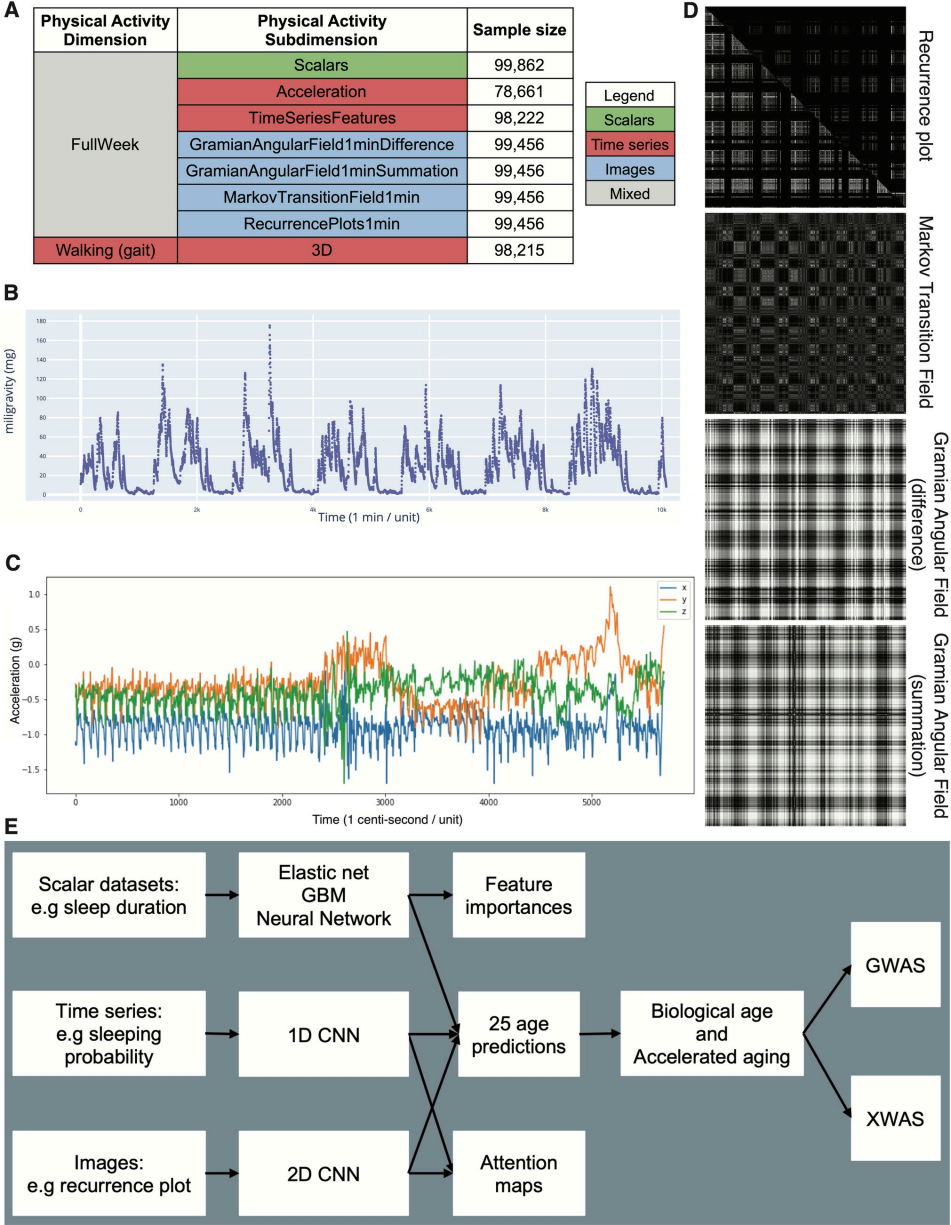
**Presentation of the datasets and overview of the analytic pipeline A - Preprocessed datasets obtained from the raw wrist accelerometer data, hierarchically ordered into physical activity dimensions and subdimensions. B - full week - Acceleration sample. Each time step corresponds to the mean acceleration over one minute. C - Gait sample, collected over one minute. D - Images generated from a raw accelerometer record. E - Overview of analytic pipeline.** D—See methods for a description of the images. For example, the recurrence plot measures the similarity between the physical activity captured at different times of the week. The axes each correspond to the full week. The upper right triangle corresponds to similarities in sleep activities, and the lower left triangle corresponds to similarities in the remaining time.

We defined physical activity dimensions by hierarchically grouping the different datasets at two different levels: we call the first level “physical activity dimensions” and the second level “physical activity subdimensions”. The complete hierarchy between the two physical activity dimensions and the eight subdimensions is described in Fig 1A. For example, one of the physical activity dimensions we investigated was “Full Week”, which is based on the participants’ physical activity over the entire week. This dimension consists of seven subdimensions: (1) the scalar features derived from the full week accelerometer data (e.g mean acceleration over the week, maximum acceleration during the weekend), (2–3) the models built on the full week time series generated from the raw data, and (4–7) the images generated from the raw data.

### Data types and preprocessing

The data preprocessing step is different for the different data modalities: demographic variables, scalar predictors, time series and images. We define scalar predictors as predictors whose information can be encoded in a single number, such as the mean acceleration, as opposed to data with a higher number of dimensions such as time series (one dimension, which is time), and images (two dimensions, which are the height and the width of the image).

### Demographic variables

First, we removed out the UKB samples for which age or sex was missing. For sex, we used the genetic sex when available, and the self-reported sex when genetic sex was not available. We computed age as the difference between the date when the participant attended the assessment center and the year and month of birth of the participant to estimate the participant’s age with greater precision. We one-hot encoded ethnicity.

### Physical activity as time series

First, we preprocessed the data to ensure we were analyzing activity that took place during the full week. To mitigate the problems associated with excessively long time series, we started with the summarized data (one lead, 0.2 Hz, field 90004) and computed the mean acceleration every minute. We discarded the samples for which less than a full week’s worth of data was available (10,080 time steps = 7 days * 24 hours * 60 minutes), and only kept the first 10080 when more data than necessary was available. This step filtered out 25,026 samples out of the initial 103,687 samples, leaving 78,661 samples. Finally, we applied a smoothing function (exponential moving average with parameter 35) to reduce the noise. We refer to this preprocessed data as “FullWeek_Acceleration” (S7 Fig).

Then, we generated a second time series (“FullWeek_TimeSeriesFeatures”) over the full week by processing the raw high frequency 3D data (field 90001). We used the “biobank Accelerometer Analysis” software [26,27] to compute the probability for each sample of performing several activities (biking, sitting/standing, vehicle, mixed, sleeping and walking) every five seconds. The software generates 113 features as an intermediate step, that it uses in a later step to classify the different activities. We computed the average for these time series every 5 seconds to leverage these features as additional channels for the FullWeek_TimeSeriesFeatures model. The final dimensions of each sample was therefore 113 channels of 7 days * 24 hours * 12 5-minutes long periods = 5,373 time steps.

We then filtered low quality samples. First, we removed 6,618 samples for which data was collected for less than a week. The summary file outputted by the software that we used to preprocess the data also contains quality indicators. We used four columns (quality-calibratedOnOwnData, quality-goodCalibration, quality-goodWearTime and quality-daylightSavingsCrossover) to filter out 8,984 low quality samples. After preprocessing the dataset, we were left with 99,862 samples.

We generated a third time series from the raw high frequency 3D data to specifically compare the walking pattern between the different participants, which we refer to as “Walking_3D”. We identified for each sample the continuous periods during which the activity was predicted by the software to be walking with a probability of 1. For each sample, we then randomly selected up to five of these sequences and extracted the corresponding 3D raw acceleration data (S8 Fig).

Finally, we generated a similar dataset to predict age as a function of physical activity during sleeping. The predictor was weak so it is not included in the main tables, but we describe our methods for researchers who would be interested in reusing this pipeline for different purposes. First we detected continuous periods of 180 and 360 minutes of sleep. Because the probability of the sleeping activity is never equal to 1 over these long periods of time, we first generated binary time series that specified every five seconds whether the activity was sleeping with a probability of 1, or not. Then we smoothed this curve by using a sliding window with a period of 30 minutes and set a threshold of 0.3 to determine the beginning and the end of the sleeping period. This allowed us to include brief periods of physical activity during the night as being part of the sleeping period. We then extracted the 3D high intensity acceleration data corresponding to the sleeping periods of 180 and 360 minutes. We extracted as many of these samples as possible for each participant. Neither the 180 minutes nor the 360 minutes hyperparameters yielded good predictors of chronological age (R-Squared = 0.016 for both models).

### Physical activity as scalar variables

We define scalar variables as a variable that is encoded as a single number, such as mean or maximum acceleration, as opposed to data with a higher number of dimensions, such as time series, and images. The complete list of scalar variables can be found in S3 Table. We did not preprocess the scalar variables, aside from the normalization that is described under cross-validation further below.

We filtered out the low-quality samples based on the quality-calibratedOnOwnData, quality-goodCalibration, quality-goodWearTime and quality-daylightSavingsCrossover indicators, as mentioned in “Physical activity as time series” above. Then we generated a total of 2,271 scalar features, from three different pipelines (S2 Table).

First, we leveraged 1,333 summary features directly outputted by the two biobank Accelerometer Analysis preprocessing softwares [27,28]. The first software [28] generated 813 features from which we filtered out 66 (S21 Table) non-biological features (e.g. features related to the calibration of the accelerometers) and kept the remaining 747 features. The second software [27] generated 896 features, from which we filtered out 67 non-biological features. We then took the union of the features generated by the two models to obtain the 1,333 features.

Second, we used the 113 time series generated by the Accelerometer Analysis software during one of its intermediate steps. For each of these time series, we computed the minimum value, the maximum value, the average value, the standard deviation, as well as the 25th, 50th and 75th percentiles, for a total of 791 features.

Finally, we built 147 custom features from six different types. (1) Summary statistics features, (2) Behavioral features, (3) Sleeping features, (4) Activity phase features, (5) Weekend features, (6) Metabolic Equivalent of Task (MET) features. The design of these features is described in detail below.

### Summary statistics features

First, we computed the average acceleration every minute for each participant, and we computed the mean, median, interquartile range, skewness, and the fifth, tenth, 25th, 75th and 90th percentile on these time series. We then computed the average acceleration over every day of the week, and we computed the following summary statistics: the mean, standard deviation, minimum, maximum, median, and the 25th and 75th percentiles. Finally, we computed the same summary statistics on the different periods of the day: (1) night (midnight to 6am), (2) morning (6am to 9am), (3) day (9am to 7pm) and (4) evening (7pm to midnight).

### Activity specific features

We computed the number of minutes spent on each activity (e.g. walking, sleeping). We then computed the distribution of time across the different activity types for each day of the week along with the standard deviation for these values. Finally, we computed the same features described under “Summary statistics features” for every physical activity. For each activity, we defined the time series on which to perform this analysis as the concatenation of periods of at least five minutes over which the activity classification remained constant.

### Sleeping features

We defined night sleep as time steps classified as sleep between 10pm and 6am, and we computed the average night sleep duration. We defined deep sleep as an uninterrupted series of timesteps classified as sleeping, and we computed the average duration of deep sleep phases for each participant. Two participants could have the same average night sleep duration of eight hours, but the first participant could have a single deep sleep phase of eight hours while the second participant could briefly wake up or move in their sleep three times throughout the night, which would read as an average deep sleep duration of only two hours. The average deep sleep duration can therefore be used to assess the quality of sleep that each participant is getting. Then, we defined a sleep session as a sleeping activity longer than twenty minutes and we computed the daily average number of sleep sessions for each participant. This feature for example captures if a participant is taking naps. Finally, we selected the longest sleep session for each participant, which represents their “night sleep”, even if some participants might be working night shifts and therefore have this “night sleep” during the day. We computed the average duration of this night’s sleep and the average time of the day at which it started for each participant.

### Physical activity features

We computed the daily average time spent walking, as well as the daily average duration of the longest continuous walk. We also computed the average time of the day associated with the first and the last peak of physical activity.

### Weekend features

We defined the weekend as the time between Friday 10pm and Sunday 11:59pm and we computed the time spent on the different activities during this period. We also computed the time at which the participants went to bed on the night of Saturday evening to Sunday morning to capture Saturday night habits.

### Metabolic Equivalent of Task (MET) features

The metabolic equivalent of a task is the rate at which a participant is expending energy when performing this task compared to when they are resting (e.g. sitting or being inactive) [29]. For example, a MET of two means that the participant is spending twice as much energy performing their current activity than they are when they are resting. Activities with a MET smaller than three (e.g. walking slowly or working at a desk) are considered to be light intensity activities. Activities with a MET between three and six qualify as moderate intensity activities (e.g. walking at a faster pace or practicing relaxing sportive activities). Activities with a MET larger than six are considered vigorous intensity activities (e.g. jogging, biking, dancing, weightlifting, competitive sportive activity). [29–33]

MET measures can be used to estimate the total energy expended over the day or the week, using the unit of MET hour. For example, if a participant performs a task with a MET of three for 40 minutes, that is equivalent to performing a task with a MET of one for two hours. In both cases, the total energy expended by the participant while practicing the activity is two MET hours.

The UKB software estimates the MET for every time step. We leveraged this column to compute the following features. First, we computed the total MET hours over the full week. Then, we computed the daily (seven values) and the hourly (24 values) MET averages and associated standard deviations. Finally, we computed the mean and the standard deviation of the MET values for low, medium and vigorous activities over the week. We defined low, medium and vigorous intensity using the aforementioned threshold values.

### Physical activity as images

Starting from the unidimensional, preprocessed acceleration average with a frequency of 0.2 Hz (field 90004), we computed the average acceleration for every minute. From there, we generated four “images” per sample for each participant.

For the first type of image, we generated a custom recurrence plot [34]. We used a sliding window of length ten minutes and stride of one minute, so the value of the pixel in row *i* and column *j* is the Euclidean distance between the vector of the 10 one-minute-average accelerations starting at the minute *i* and the vector of the 10 one-minute-average accelerations starting at the minute *j* and stored them as floats. We generated two recurrence plots. One using only the time steps for which the probability of the participant sleeping was 1, and one using only the remaining time steps. Because recurrence plots are symmetrical, we merged these two recurrence plots into a single image, in which the upper right triangle corresponds to the sleeping timesteps, and the lower left triangle corresponds to the remaining time steps (S6 and S7 Figs).

Second, we generated a Markov transition fields image (n_bins = 8), a Gramian angular difference fields image and a Gramian angular summation field image [35] respectively using the MarkovTransitionField and the GramianAngularField functions of the pyts python package [36]. Finally, we resized all the images to be of size 316*316. A sample of these three images can be found in S6 and S7 Figs.

We generated other images that we ended up not using for the analysis. We will describe them here as knowing that they were poor age predictors might save time for researchers considering similar approaches. First, we generated the recurrence plots using the average acceleration over periods of 32 minutes instead of one minute, which generated images of dimension 316*316 directly, saving the resizing step. We did not observe significant differences between the two methods when we explored this possibility, so we discarded it. We also applied the binary filter traditionally used for recurrence plots, by setting to one the value for the pairs of points that were in the 95th percentile for proximity. We found that the performances were similar, with a possible advantage for using float values. Finally, we also applied these four methods (recurrence plot, Markov transition field and Gramian angular summation and Gramian angular difference) to selected segments of 3D data for the walking and the sleeping activities, possibly capturing the recurrence patterns in the gait, for example. During our preliminary analysis and early exploration of the hyperparameters, we found that none of these 3D data-based plots yielded R-Squared values higher than 0.05.

### Machine learning algorithms

For scalar datasets, we used elastic nets, gradient boosted machines [GBMs] and fully connected neural networks. For times series, images and videos we used one-dimensional, two-dimensional, and three-dimensional convolutional neural networks, respectively.

### Algorithms that input scalar variables

We used three different algorithms to predict age from scalar variables (see S3 Table for list of scalar physical activity variables) Elastic Nets [EN] (a regularized linear regression that represents a compromise between ridge regularization and LASSO regularization), Gradient Boosted Machines [GBM] (LightGBM implementation [37]), and Neural Networks [NN]. The choice of these three algorithms represents a compromise between interpretability and performance. Linear regressions and their regularized forms (LASSO [38], ridge [39], elastic net [40]) are highly interpretable using the regression coefficients but are poorly suited to leverage non-linear relationships or interactions between the features and therefore tend to underperform compared to the other algorithms. In contrast, neural networks [41,42] are complex models, which are designed to capture non-linear relationships and interactions between the variables. However, tools to interpret them are limited [43] so they are closer to a “black box”. Tree-based methods such as random forests [44], gradient boosted machines [45] or XGBoost [46] represent a compromise between linear regressions and neural networks in terms of interpretability. They tend to perform similarly to neural networks when limited data is available, and the feature importances can still be used to identify which predictors played an important role in generating the predictions. However, unlike linear regression, feature importances are always non-negative values, so one cannot interpret whether a predictor is associated with older or younger age. We also performed preliminary analyses with other tree-based algorithms, such as random forests [44], vanilla gradient boosted machines [45] and XGBoost [46]. We found that they performed similarly to LightGBM, so we only used this last algorithm as a representative for tree-based algorithms in our final calculations.

### Algorithms that input time series accelerometer data

We developed a different architecture for the three time series extracted from the wrist accelerometers data.

### Architecture for raw acceleration data over the full week

For the model based on the acceleration data across the full week (FullWeek acceleration), we used the architecture from the paper "Extracting biological age from biomedical data via deep learning: too much of a good thing?" [9]. The architecture of the model is described in S10 Fig and S11 Fig, and is constituted of two blocks: (1) a convolutional block and (2) a dense block. These two blocks form a seven layers deep neural network, preceded by a batch normalization. The inputs for the convolutional block are time series with a single channel and 10,080 time steps. The convolutional block consists of four one-dimensional convolutional layers with respectively 64, 32, 32 and 32 filters, a kernel size of respectively 128, 32, eight and eight, and a stride of one. Every convolutional layer is zero-padded and followed by a max pooling with a size of respectively four, four, three and three, and with a stride of two. The output of the convolutional block is inputted into the dense block which consists of three dense layers. The first two dense layers have respectively 256 and 128 nodes and are using dropout. The first six layers of the architecture use kernel and bias regularization, and use ReLU as their activation function. The seventh layer is a dense layer with size one and with a linear activation function to predict chronological age.

During our preliminary analysis, we tested a second architecture, from the article “Deep Learning using Convolutional LSTM estimates Biological Age from Physical Activity” [10]. This architecture uses both convolutional layers and LSTM layers and takes as input the data formatted as a 3-dimensional data frame of dimensions 7x24x60. The first dimension is for the days of the week, the second for the hours of the day and the third for the minutes of the hour. This architecture explained 33% of the variance in chronological age but was outperformed by the first model described above and took significantly longer to train. As a result, we did not select this second architecture for our final pipeline.

### Architecture for features extracted from the acceleration data over the full week

To analyze the 113 time series of features extracted from the raw acceleration data, we built a 14 layers deep architecture which can be found in S12 Fig and S13 Fig. The architecture consists of a convolutional block and a dense block. The inputs for the convolutional block are time series with 113 channels and 2,016 time steps. The convolutional block comprises nine one-dimensional convolutional layers with the number of filters for each layer doubling with every layer, starting from 64 and with a maximum number of filters of 4,096 (64, 128, 256, 512, 1,024, 2,048, 4,096, 4,096, 4,096). Each convolutional layer is zero-padded and followed by a one-dimensional max pooling layer of size two and stride 2. The output of the convolutional block is converted to 4,096 scalar features using a global max pooling over each of the 4,096 filters. A single layer neural network (409 nodes) is parallelly taking as input the 113 scaling factors for the 113 normalized time series that were inputted in the convolutional block. The 4,096 convolutional based features are concatenated with the 409 scaling factor-based features, and the resulting 4,505 scalar features are inputted in the dense block. The dense block is composed of five dense layers with respectively 1,024, 1,024, 1,024, 512 and one node(s). All dense layers, except for the last one, use dropout. The last layer uses linear activation to predict chronological age. A single layer (25 nodes) neural network is trained on sex and ethnicity, and its output is concatenated with the output of the second dense layer before being inputted in the third dense layer. All the layers of the architecture aside from the final layer use batch normalization, kernel and bias regularization, and ReLU for their activation function.

### Architecture for three-dimensional high frequency walking data

We built a 13 layers-deep convolutional architecture to analyze the walking data. This architecture can be found in S14 Fig and S15 Fig. The architecture is highly similar to the one described above. It comprises a convolutional block and a dense block. The inputs for the convolutional block are time series with three channels and 5,700 time steps (5.7 seconds of 3-dimensional acceleration). The convolutional block consists of eight one-dimensional convolutional layers with the number of filters for each layer doubling with every layer, starting from 32 and with a maximum number of filters of 2,048 (32, 64, 128, 256, 512, 1,024, 2,048, 2,048). All convolutional kernels are zero-padded, of size three and with a stride of one. Each convolutional layer uses batch normalization, ReLU as activation function, and is followed by a one-dimensional max pooling layer of size two and stride 2. The output of the convolutional block is converted to 2,048 scalar features using a global max pooling over each of the 4,096 filters. A single layer neural network (204 nodes) is parallelly taking as input the 3 scaling factors for the x-axis, y-axis and z-axis normalized time series that were inputted in the convolutional block (the three dimensions of the accelerometer). The 4,096 convolutional based features are concatenated with the 204 scaling factor-based features, and the resulting 2,252 scalar features are inputted in the dense block. The dense block is composed of five dense layers with respectively 1,024, 1,024, 1,024, 512 and 1 nodes. The first four dense layers use dropout. The last layer uses linear activation to predict chronological age. A single layer (25 nodes) neural network is trained on sex and ethnicity, and its output is concatenated with the output of the second dense layer before being inputted in the third dense layer. All layers, except for the last one, use batch regularization, kernel and bias regularization, and use ReLU as their activation function. We used Adam [47] as the compiler for all the time series models.

### Algorithms that input image data

#### Convolutional Neural Networks Architectures

We used transfer learning [48–50] to leverage two different convolutional neural networks [51] [CNN] architectures pre-trained on the ImageNet dataset [52–54] and made available through the python Keras library [55]: InceptionV3 [56] and InceptionResNetV2 [57]. We considered other architectures such as VGG16 [58], VGG19 [58] and EfficientNetB7 [59], but found that they performed poorly and inconsistently on our datasets during our preliminary analysis and we therefore did not train them in the final pipeline. For each architecture, we removed the top layers initially used to predict the 1,000 different ImageNet images categories. We refer to this truncated model as the “base CNN architecture”.

We added to the base CNN architecture what we refer to as a “side neural network”. A side neural network is a single fully connected layer of 16 nodes, taking the sex and the ethnicity variables of the participant as input. The output of this small side neural network was concatenated to the output of the base CNN architecture described above. This architecture allowed the model to consider the features extracted by the base CNN architecture in the context of the sex and ethnicity variables. For example, the presence of the same physical activity feature can be interpreted by the algorithm differently for a male and for a female. We added several sequential fully connected dense layers after the concatenation of the outputs of the CNN architecture and the side neural architecture. The number and size of these layers were set as hyperparameters. We used ReLU [60] as the activation function for the dense layers we added, and we regularized them with a combination of weight decay [61,62] and dropout [63], both of which were also set as hyperparameters. Finally, we added a dense layer with a single node and linear activation to predict age.

#### Compiler

The compiler uses gradient descent [64,65] to train the model. We treated the gradient descent optimizer, the initial learning rate and the batch size as hyperparameters. We used mean squared error [MSE] as the loss function, root mean squared error [RMSE] as the metric and we clipped the norm of the gradient so that it could not be higher than 1.0 [66].

We defined an epoch to be 32,768 images. If the training loss did not decrease for seven consecutive epochs, the learning rate was divided by two. This is theoretically redundant with the features of optimizers such as Adam, but we found that enforcing this manual decrease of the learning rate was sometimes beneficial. During training, after each image has been seen once by the model, the order of the images is shuffled. At the end of each epoch, if the validation performance improved, the model’s weights were saved.

We defined convergence as the absence of improvement on the validation loss for 15 consecutive epochs. This strategy is called early stopping [66] and is a form of regularization. We requested the GPUs on the supercomputer for ten hours. If a model did not converge within this time and improved its performance at least once during the ten hours period, another GPU was later requested to reiterate the training, starting from the model’s last best weights.

### Training, tuning and predictions

We split the entire dataset into ten data folds. We then tuned the models built on scalar data, on time series, on images and on videos using four different pipelines. For scalar data-based models, we performed a nested-cross validation. For time series-based and images-based models, we manually tuned some of the hyperparameters before performing a simple cross-validation. We describe the splitting of the data into different folds and the tuning procedures in greater detail in the Supplementary.

### Interpretability of the machine learning predictions

To interpret the models, we used the regression coefficients for the elastic nets, the feature importances for the GBMs, a permutation test for the fully connected neural networks, and attention maps (saliency and Grad-RAM) for the convolutional neural networks (Supplementary Methods).

### Ensembling to improve prediction and define aging dimensions

We built a three-level hierarchy of ensemble models to improve prediction accuracies. At the lowest level, we combined the predictions from different algorithms on the same aging subdimension. For example, we combined the predictions generated by the elastic net, the gradient boosted machine and the neural network on the scalar predictors (subdimension). At the second level, we combined the predictions from different subdimensions of a unique dimension. For example, we combined the models built on the seven subdimensions (one scalar-based model, two time series-based models and four image-based models) of the “Full week” dimension into an ensemble prediction. Finally, at the highest level, we combined the predictions from the two physical activity dimensions into a general biological age prediction. The ensemble models from the lower levels are hierarchically used as components of the ensemble models of the higher models. For example, the ensemble models built by combining the algorithms at the lowest level for each of the Full Week subdimensions are leveraged when building the general, overarching ensemble model.

We built each ensemble model separately on each of the ten data folds. For example, to build the ensemble model on the testing predictions of the data fold #1, we trained and tuned an elastic net on the validation predictions from the data fold #0 using a 10-folds inner cross-validation, as the validation predictions on fold #0 and the testing predictions on fold #1 are generated by the same model (see Methods - Training, tuning and predictions—Images—Scalar data—Nested cross-validation; Methods—Training, tuning and predictions—Images—Cross-validation). We used the same hyperparameters space and Bayesian hyperparameters optimization method as we did for the inner cross-validation we performed during the tuning of the non-ensemble models.

To summarize, the testing ensemble predictions are computed by concatenating the testing predictions generated by ten different elastic nets, each of which was trained and tuned using a 10-folds inner cross-validation on one validation data fold (10% of the full dataset) and tested on one testing fold. This is different from the inner-cross validation performed when training the non-ensemble models, which was performed on the “training+validation” data folds, so on 9 data folds (90% of the dataset).

### Evaluating the performance of models

We evaluated the performance of the models using three different measures, including R-Squared [R^2^], root mean squared error [RMSE], and mean absolute error [MAE]. We computed a confidence interval on the performance metrics in two different ways. First, we computed the standard deviation between the different data folds. The test predictions on each of the ten data folds are generated by ten different models, so this measure of standard deviation captures both model variability and the variability in prediction accuracy between samples. Second, we computed the standard deviation by bootstrapping the computation of the performance metrics 1,000 times. This second measure of variation does not capture model variability but evaluates the variance in the prediction accuracy between samples.

### Biological age and bias in the predicted age

We observed a bias in the predicted, or biological age. For each model, participants on the older end of the chronological age distribution tend to be predicted younger than they are. Symmetrically, participants on the younger end of the chronological age distribution tend to be predicted older than they are. This bias does not seem to be biologically driven. Rather it seems to be statistically driven, as the same 60-year-old individual will tend to be predicted younger in a cohort with an age range of 60–80 years, and to be predicted older in a cohort with an age range of 40–60. We ran a linear regression on the residuals as a function of age for each model and used it to correct each prediction for this bias.

After defining biological age as the corrected prediction, we defined accelerated aging as the corrected residuals. For example, a 60-year-old with a predicted age of 70 years old after correction for the bias in the residuals is estimated to have a biological age of 70 years, and an accelerated aging of ten years.

It is important to understand that this step of correction of the predictions and the residuals takes place after the evaluation of the performance of the models but precedes the analysis of the biological ages properties.

### Genome-wide association of accelerated aging

The UKB contains genome-wide genetic data for 488,251 of the 502,492 participants [67] under the hg19/GRCh37 build.

We used the average accelerated aging value over the different samples collected over time (see Supplementary—Models ensembling—Generating average predictions for each participant). Next, we performed genome wide association studies [GWASs] to identify single-nucleotide polymorphisms [SNPs] associated with accelerated aging using BOLT-LMM [22,23] and estimated the the SNP-based heritability for each of our biological age phenotypes, and we computed the genetic pairwise correlations between dimensions using BOLT-REML [24]. We used the v3 imputed genetic data to increase the power of the GWAS, and we corrected all of them for the following covariates: age, sex, ethnicity, the assessment center that the participant attended when their DNA was collected, and the 20 genetic principal components precomputed by the UKB. We used the linkage disequilibrium [LD] scores from the 1,000 Human Genomes Project [68, 69]. To avoid population stratification, we performed our GWAS on individuals with White ethnicity.

### Identification of SNPs associated with accelerated aging

We identified the SNPs associated with accelerated aging using the BOLT-LMM [22,23] software (p-value of 5e-8). The sample size for the genotyping of the X chromosome is one thousand samples smaller than for the autosomal chromosomes. We therefore performed two GWASs for each aging dimension. (1) excluding the X chromosome, to leverage the full autosomal sample size when identifying the SNPs on the autosome. (2) including the X chromosome, to identify the SNPs on this sex chromosome. We then concatenated the results from the two GWASs to cover the entire genome, at the exception of the Y chromosome. As we described previously, we used the Functional Mapping and Annotation (FUMA) software [70] to identify (1) the loci associated with each of the physical activity aging, and the (2) nearest protein coding genes. To identify loci, SNPs are filtered that have a GWAS-level of significance (in our study, p < 5e-8). SNPs that are GWA-significant and have a r^2^ greater than 0.6 are candidate SNPs for a locus; other SNPs are considered independent. The SNP with the lowest p-value and independent of other SNPs at a r^2^ less than 0.1 is the “lead SNP”. To identify closest protein coding genes, FUMA uses ANNOVAR [71], to positionally map SNPs. A quality control of our GWAS is in the Supplementary Methods.

### Heritability and genetic correlation

We estimated the heritability of the accelerated aging dimensions using the BOLT-REML [24] software. We included the X chromosome in the analysis and corrected for the same covariates as we did for the GWAS.

### Non-genetic correlates of accelerated aging

We identified non-genetically measured (i.e factors not measured on a GWAS array) correlates of each aging dimension, which we classified in six categories: biomarkers, clinical phenotypes, diseases, family history, environmental, and socioeconomic variables. We refer to the union of these association analyses as an X-Wide Association Study [XWAS]. (1) We define as biomarkers the scalar variables measured on the participant, which we initially leveraged to predict age (e.g. blood pressure, S5 Table). (2) We define clinical phenotypes as other biological factors not directly measured on the participant, but instead collected by the questionnaire, and which we did not use to predict chronological age. For example, one of the clinical phenotypes categories is eyesight, which contains variables such as “wears glasses or contact lenses”, which is different from the direct refractive error measurements performed on the participants, which are considered “biomarkers” (S8 Table). (3) Diseases include the different medical diagnoses categories listed by UKB (S11 Table). (4) Family history variables include illnesses of family members (S13 Table). (5) Environmental variables include alcohol, diet, electronic devices, medication, sun exposure, early life factors, medication, sun exposure, sleep, smoking, and physical activity variables collected from the questionnaire (S14 Table). (6) Socioeconomic variables include education, employment, household, social support and other sociodemographics (S17 Table). We provide information about the preprocessing of the XWAS in the Supplementary Methods.

## Results

### Overview

We analyzed 115,456 one week-long wrist accelerometer records collected from 103,680 UK Biobank participants aged 43.5 to 79.0 years (S2 Fig, Fig 1). First, we preprocessed the raw one week-long 100Hz time series (60,480,000 time steps) into 2,271 scalar variables (e.g average acceleration, number of hours sleeping, number of hours exercising, S2 Table), 113 time series with one time step per minute (e.g.probability of being asleep, maximum physical activity, minimum physical activity) and four images (recurrence plot, Markov transition field and Gramian angular field of difference and summation). For each sample, we also extracted a one minute-long 100Hz sample for which the participant is walking (Fig 1C). This dataset, referred to as “Walking” (Fig 1A) allows us to test whether age can be predicted from the gait, in contrast to the other datasets who summarize the activity pattern over the full week.

### We predicted chronological age within five years

**Fig 2:**
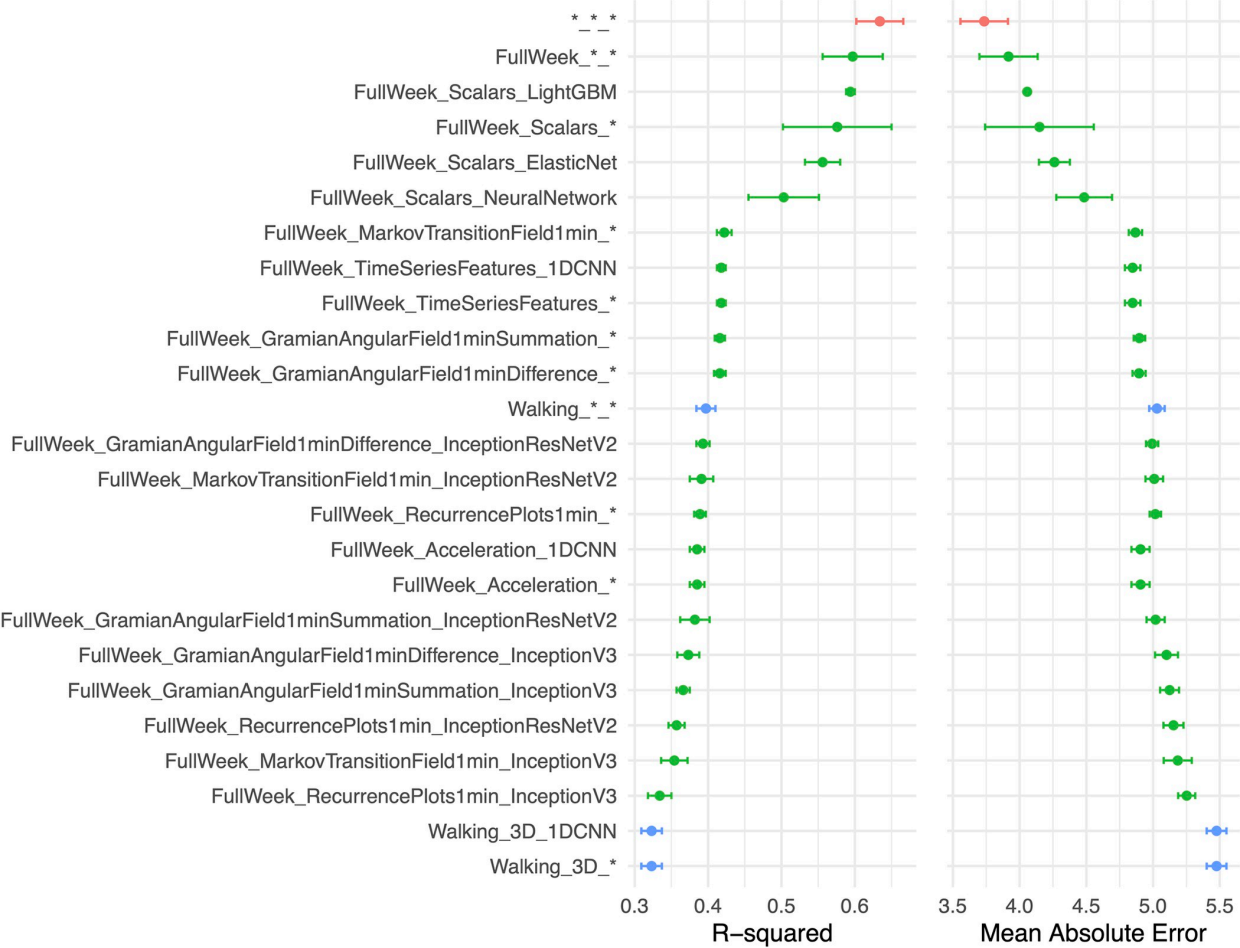
Chronological age prediction performance. **A.) R2 (R-squared) and B.) Mean Absolute Error (MAE).** Model name contain the duration (e.g., full week), data type (e.g., Scalars or Time Series), and model algorithm type (e.g., LightGBM). * represents ensemble model. Red point is the overall ensemble across all models, green points denote the measured physical activity for the full week as inputs, and blue points denote the walking or gait data as inputs.

We deployed different machine learning approaches for each type of data modality (Fig 2, S1 Table). We predicted age from scalar variables using an elastic net, a gradient boosted machine [GBM] and a shallow, fully connected neural network. We predicted age from time-series data using one-dimensional convolutional neural networks [CNNs]. We predicted age from images using two-dimensional CNNs.

We hierarchically ensembled the models and predicted age with a R-Squared [R^2^] of 63.5±3.2% and a mean absolute error [MAE] of 3.7±0.2 years (S2 Fig). In terms of the different physical activity “dimensions” (full week activities vs. gait), we predicted age with respective R^2^ values of 59.7±4.1% and 39.7±1.3%. Accelerated aging, as defined by the full week activity pattern, was 0.266±.004 correlated with walking-based or gait-based accelerated aging (S6 Fig). For the models built on the full week data, the best performance was obtained from the GBM trained on the scalar features (R^2^ = 59.4±0.6%).

We report the feature importances for the elastic net, the GBM and the neural network in S3 Table. We generated attention maps for the time series (S5 Fig) and the images (S3 Fig). More examples can be respectively be found at https://www.multidimensionality-of-aging.net/model_interpretability/time_series and https://www.multidimensionality-of-aging.net/model_interpretability/images under “Physical Activity”.

### Genetic factors and heritability of accelerated physical activity-derived aging

We defined the biological age of each participant as their predicted age by the model, and accelerated aging as the difference between their chronological and biological age. We then performed a genome wide association study [GWAS] and found accelerated aging to be 12.3±0.9% GWAS-heritable. Information on the quality control of the GWAS can be found in the Supplementary Methods.

**Fig 3:**
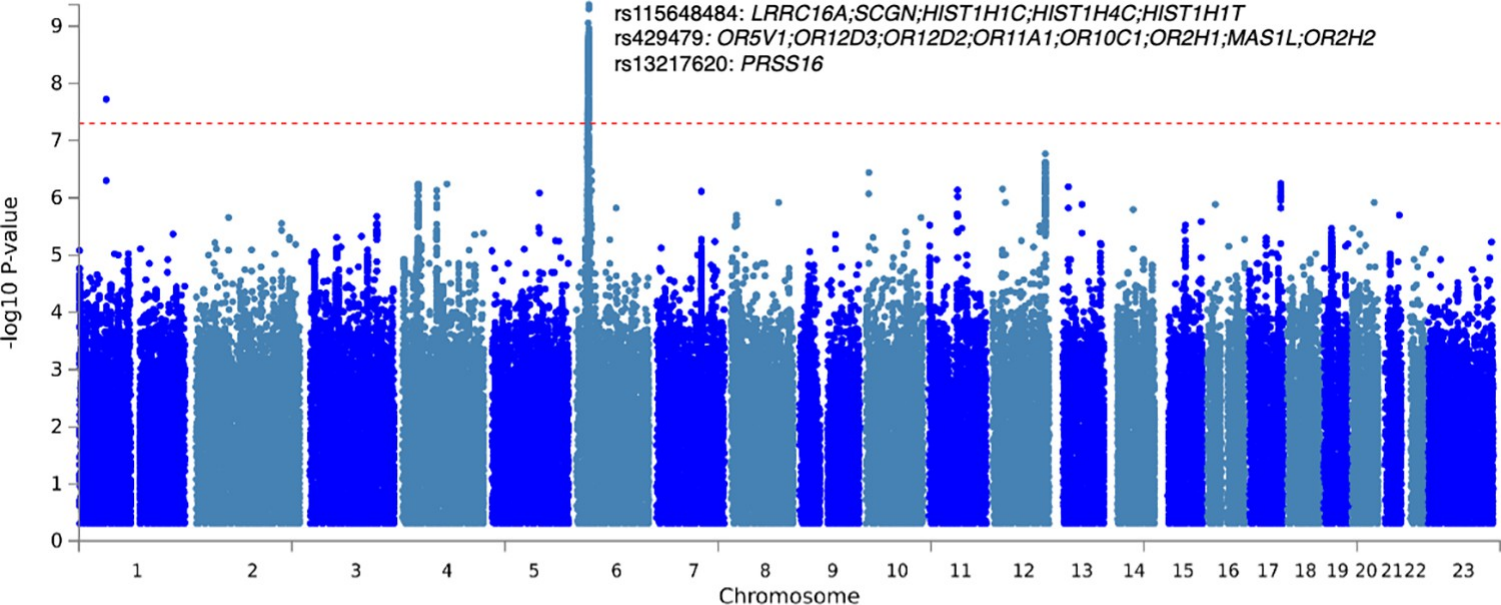
Genome-wide association study on physical activity based accelerated aging: negative log10 (p-value) vs. chromosomal position. Dotted line denotes 5e-8. Genomic control inflation factor (lambda) was 1.04. 3 of the ten11 SNPs found in chromosome 6 are labeled.

The GWAS highlighted a peak on chromosome 6 (Fig 3), consisting of ten independent associations that were in a region proximal to many histone genes (e.g., *HISTH1C*, *HIST14C*) and olfactory-related genes (e.g., *OR5V1*) [72]. See S4 Table for summary statistics and S4 Fig for an in-depth view of associations in LocusZoom format.

### Non-genetic factors correlated with accelerated aging

We performed a systematic correlation study, or X-wide association study [XWAS], to identify biomarkers (S5 Table), clinical phenotypes (S8 Table), diseases (S11 Table), environmental (S14 Table) and socioeconomic (S17 Table) variables correlated with accelerated aging. We found no association between accelerated aging and family history variables. We summarize our findings below. The exhaustive results can be found in S20 Table and explored at https://www.multidimensionality-of-aging.net/xwas/univariate_associations. Most of the correlations were modest in size (median absolute value of partial correlation was 0.01, and the 99th percentile was 0.23).

### Biomarkers correlated with accelerated aging

The three biomarker categories most correlated with accelerated aging are blood pressure, anthropometry, and pulse wave analysis (a measure of arterial stiffness) biomarkers (S6 Table). Specifically, 100.0% of blood pressure biomarkers are correlated with accelerated aging, with the three largest associations being with pulse rate (correlation = .082), systolic blood pressure (correlation = .041), and diastolic blood pressure (correlation = .024). 62.5% of anthropometry biomarkers are correlated with accelerated aging, with the three largest associations being with waist circumference (correlation = .114), hip circumference (correlation = .094), and weight (correlation = .092). 50.0% of arterial stiffness biomarkers are correlated with accelerated aging, with the three largest associations being with pulse rate (correlation = .078), position of the pulse wave notch (correlation = .063), and position of the shoulder on the pulse waveform (correlation = .046).

Conversely, the three biomarker categories most correlated with decelerated aging are symbol digit substitution (a cognitive test), hand grip strength and spirometry (S7 Table). Specifically, 100.0% of symbol digit substitution biomarkers are correlated with decelerated aging, with the two associations being with the number of symbol digit matches attempted (correlation = .096) and the number of symbol digit matches made correctly (correlation = .088). 100.0% of hand grip strength biomarkers are correlated with decelerated aging, with the two associations being with right hand grip strength (correlation = .088) and left hand grip strength (correlation = .086). 100.0% of spirometry biomarkers are correlated with decelerated aging, with the three associations being with forced expiratory volume in one second (correlation = .074), forced vital capacity (correlation = .071), and peak expiratory flow (correlation = .057).

### Clinical phenotypes correlated with accelerated aging

The three clinical phenotype categories most correlated with accelerated aging are chest pain, breathing and claudication (S9 Table). Specifically, 100.0% of chest pain clinical phenotypes are correlated with accelerated aging, with the three largest associations being with chest pain or discomfort walking normally (correlation = .045), chest pain or discomfort (correlation = .043), and chest pain due to walking ceases when standing still (correlation = .042). 100.0% of breathing clinical phenotypes are correlated with accelerated aging, with the two associations being with shortness of breath walking on level ground (correlation = .087) and wheeze or whistling in the chest in the last year (correlation = .032). 53.8% of claudication clinical phenotypes are correlated with accelerated aging, with the three largest associations being with leg pain on walking (correlation = .069), leg pain when walking uphill or hurrying (correlation = .069), and leg pain when standing still or sitting (correlation = .068).

Conversely, the three clinical phenotype categories most correlated with decelerated aging are cancer screening (most recent bowel cancer screening: correlation = .028), mouth health (no mouth or teeth dental problem: correlation = .023) and general health (no weight change during the last year: correlation = .023). (S10 Table)

### Diseases correlated with accelerated aging

The three disease categories most correlated with accelerated aging are cardiovascular, a category encompassing various diseases (category R, see S11 Table) and gastrointestinal diseases (S12 Table). Specifically, 40.3% of cardiovascular diseases are correlated with accelerated aging, with the three largest associations being with hypertension (correlation = .098), chronic ischaemic heart disease (correlation = .051), and atrial fibrillation and flutter (correlation = .046). 39.8% of the various diseases category are correlated with accelerated aging, with the three largest associations being with pain in the throat and chest (correlation = .041), nausea and vomiting (correlation = .037), and abdominal and pelvic pain (correlation = .036). 34.7% of gastrointestinal diseases are correlated with accelerated aging, with the three largest associations being with gastro-intestinal reflux disease (correlation = .041), diaphragmatic hernia (correlation = .037), and cholelithiasis (correlation = .032).

### Environmental variables correlated with accelerated aging

The three environmental variable categories most correlated with accelerated aging are sleep, sun exposure and smoking (S15 Table). Specifically, 57.1% of sleep variables are correlated with accelerated aging, with the three largest associations being with napping during the day (correlation = .095), sleeplessness/insomnia (correlation = .063), and daytime dozing/sleeping-narcolepsy (correlation = .058). 30.0% of sun exposure variables are correlated with accelerated aging, with the three largest associations being with looking approximately one’s age (correlation = .055), not knowing whether we look younger or older in terms of facial aging (correlation = .046), and time spent outdoors in the summer (correlation = .043). 29.2% of smoking variables are correlated with accelerated aging, with the three largest associations being with number of cigarettes currently smoked daily (correlation = .054), currently smoking tobacco on most or all days (correlation = .054), and difficulty not smoking for one day (correlation = .053).

Conversely, the three environmental variable categories most correlated with decelerated aging are electronic devices, physical activity (questionnaire) and smoking (S16 Table). Specifically, 40.0% of electronic devices variables are correlated with decelerated aging, with the three largest associations being with weekly usage of mobile phone in the last three months (correlation = .089), length of mobile phone use (correlation = .073), and hands-free device/speakerphone use with mobile phone in the last three months (correlation = .064). 37.1% of physical activity questionnaire variables are correlated with decelerated aging, with the three largest associations being with usual walking pace (correlation = .141), types of physical in the last four weeks: strenuous sports (correlation = .113), and frequency of strenuous sports in the last four weeks (correlation = .102). 25.0% of smoking variables are correlated with decelerated aging, with the three largest associations being with time from waking to first cigarette (correlation = .057), age started smoking in current smokers (correlation = .054), and not currently smoking tobacco (correlation = .045).

### Socioeconomic variables correlated with accelerated aging

The three socioeconomic variable categories most correlated with accelerated aging are sociodemographics, social support and household (S18 Table). Specifically, 28.6% of sociodemographic variables are correlated with accelerated aging, with the two associations being with private healthcare (correlation = .043) and receiving attendance allowance (correlation = .033). 25.0% of social support variables are correlated with accelerated aging, with the two associations being with leisure/social activities: religious group (correlation = .084) and not having social activities (correlation = .040). 15.0% of household variables are correlated with accelerated aging, with the three largest associations being with owning one’s accommodation (correlation = .091), gas or solid fuel cooking/heating: a gas fire that you use regularly in winter time (correlation = .043), and renting one’s accommodation from local authority, local council or housing association (correlation = .043).

Conversely, the three socioeconomic variable categories most correlated with decelerated aging are employment, social support and household (S19 Table). Specifically, 38.1% of employment variables are correlated with decelerated aging, with the three largest associations being with length of working week for main job (correlation = .238), current employment status: in paid employment or self-employed (correlation = .210), and frequency of traveling from home to job workplace (correlation = .204). 25.0% of social support variables are correlated with decelerated aging, with the two associations being with attending sports club or gym (correlation = .087) and attending a pub or social club (correlation = .049). 15.0% of household variables are correlated with decelerated aging, with the three largest associations being with total household income before tax (correlation = .142), owning one’s accommodation with a mortgage (correlation = .115), and number of vehicles in household (correlation = .063).

## Discussion

In this paper, we aimed to predict age from wrist accelerometer records. We trained our algorithm on chronological age and we claim that the predicted chronological age is an indicator of “biological age”. The difference between the predicted chronological age and actual chronological age is known as “accelerated age”.

We predicted age from wrist accelerometer records (R^2^ = 63.4; MAE = 3.7years). Specifically, we predicted age from both the full week activity pattern (R^2^ = 59.7) and the gait (R^2^ = 39.7) of the participants, and found the two accelerated aging phenotypes derived from these models to be mildly correlated (.266±.004). This suggests that both weekly physical activity patterns and gait are changing with age, but that a single individual might for example have a gait more typical of someone younger than them, despite having a weekly physical activity pattern more typical of someone older than them.

We performed a GWAS and found that accelerated aging is partly heritable (h_g^2^ = 12.3±0.9%) with ten SNPs in chromosome six within distance of many genes [73]. One locus was in close proximity to genes that encode play a role in regulation of gene expression, histones (e.g., *HIST1H4C*), large proteins that serve as a spool to wrap DNA. Epigenetic modifications, such as those to histones, have long been hypothesized as a mechanism by which exercise and physical activity may exert its biological function [74,75]. We also found, again in the same general chromosomal location, variants in proximity to olfactory-related genes (e.g., *OR5V1*), among the largest genes in the body. Olfactory function has been associated with other aspects of aging, particularly in neurodegenerative phenotypes and diseases (e.g., Alzheimer’s Disease, see review [76]). In observational/cross-sectional studies, age-related olfactory decline has been implicated in dosage of exercise [77]; however, it is a challenge to ascribe causality due to the cross-sectional study design.

We identified a vast, but modest, list of variables correlated with accelerated aging which points to behavioral and non-genetic factors associated with aging, and/or a list of potential confounds. Accelerated aging is correlated with biomarkers, clinical phenotypes linked to cardiovascular (blood pressure, arterial stiffness, electrocardiogram, pulse wave analysis, chest pain, cardiovascular diseases), pulmonary (spirometry, breathing, blood count, pulmonary diseases, anaemia), musculoskeletal (hand grip strength, heel bone densitometry, claudication, joint pain, arthritis, falls, fractures) and metabolic health (anthropometry, impedance, blood biochemistry, diabetes, obesity and other metabolic diseases). These correlations with cardio-respiratory [78,79], metabolic [80] and musculoskeletal health [81] help to confirm the biological relevance of accelerated aging as measured by physical activity.

Additionally, accelerated aging is correlated with biomarkers, clinical phenotypes and diseases in physiological dimensions such as cognitive function, brain MRI features, mental health, brain diseases, hearing, eyesight, eye diseases, gastro-intestinal diseases. These correlations could be explained by their indirect effect of the physical activity pattern of the participant. Another explanation is that accelerated cardiovascular, musculoskeletal and metabolic aging is part of a general aging process, and that accelerated aging as captured by an accelerometer is correlated with accelerated aging in all organs, even the ones that are not directly involved in physical activity. This second hypothesis is supported by the correlation between wrist accelerometer-derived accelerated aging and general health factors (e.g general health rating, long-standing illness disability or infirmity, personal history of medical treatment or disease). Interestingly, decelerated aging is also associated with looking younger than one’s age (correlation = .084). We explored the connection between wrist accelerometer-derived aging and aging of other organ systems in a different paper [82].

In terms of environmental and/or non-genetic exposures, accelerated aging is correlated with self-reported sleep, time spent outdoors, and time spent on electronic devices. These correlations are expected since these features were leveraged by the model to predict chronological age in the first place. Accelerated aging is also correlated with smoking, dietary factors (e.g processed meat intake), alcohol intake (e.g frequency) and medication (e.g taking a cholesterol-lowering medication) [83]. We also found that being breastfed as a baby and being comparatively shorter at age ten is associated with decelerated aging. During youth and early adulthood, taller height is associated with higher blood pressure. Height is reported to be negatively correlated with blood pressure [83], but in the UK Biobank cohort, the correlation is positive until approximately sixty-five years [84, 85]. Socioeconomic adversity and status (sociodemographics, social support, household, education, employment) are negatively associated with accelerated aging, reflecting the literature.

The systematic correlational study also highlighted candidate lifestyle interventions to slow aging (e.g for diet: salad/raw vegetable intake, eating butter as opposed to margarine), but an important limitation of our study is that UKB is an observational study and correlation does not imply causation. A future direction could be to leverage participants with repeated measurements to test whether a change in lifestyle (e.g change of diet) between the two measurements led to slower biological aging.

We have extensively compared our physical activity-derived age predictors with the ones already existing in the literature in the supplementary (S21 Table and S1 Text). First, Pyrkov et al. predicted chronological age on 7,454 samples with an age range of 6–84 years and obtained a R^2^ value of approximately 56% (Pearson correlation: 75%) and a RMSE of 14.0 years using a CNN [8]. Second, Rahman and Adjeroh predicted chronological age on 4,268 samples with an age range of 18–84 years with a RMSE of 15.74 years [10]. Our ensemble model predicted chronological age using wrist accelerometer data with a RMSE of 4.71 years, a MAE of 3.7 years, and a R^2^ value of 63.4. Our RMSE is therefore approximately three times smaller than the RMSEs reported by Pyrkov et al. (RMSE = 14.0) and Rahman and Adjeroh (RMSE = 15.74), but our better performance is in part driven by the narrower age range covered by UKB (UKB: 43–79 years; Pyrkov et al.: 6–84 years; Rahman and Adjeroh: 18–84 years). Our model also outperformed the two NHANES based models in terms of R^2^ values (our model: 63.5±0.2%; Pyrkov et al.: 56%; Rahman and Adjeroh: 38.4%) despite being built on a narrower age range.

We hypothesize that four factors can explain our better performance. (1) The sample size we leveraged from the UKB dataset after preprocessing and filtering the samples is twelve to twenty times larger the sample sizes that Pyrkov et al. and Rahman and Adjeroh could extract from the NHANES dataset (UKB: 90,000–100,000, NHANES: 4,268–7,454). (2) Unlike UKB, NHANES accelerometer data is a frequency measure that does not capture the absolute value of the acceleration. UKB data is also available at two different sampling frequencies (0.2 Hz and 100 Hz). (3) We found that our best predictive non-ensemble model was the GBM we trained on 2,271 scalar features (R^2^ = 59.4±0.2%, RMSE = 5.11±0.1 years). In contrast, our best non-ensemble model built on time series gave a R^2^ value of 41.8±0.3% and a RMSE of 6.11±0.1 years. The models built by Pyrkov et al. and Rahman and Adjeroh rely on time series data compared to our scalar based model. (4) Building ensemble models increased our R^2^ value from R^2^ = 59.4±0.2% to 63.5±0.2%. Both Pyrkov et al. and Rahman and Adjeroh built several models but since, to our knowledge, they did not create an ensemble, we report the performances of their non-ensembled models. (5) We built both the CNN architecture designed by Pyrkov et al. and the ConvLSTM* architecture designed by Rahman and Adjeroh, which is their best performing architecture. We found that the architecture designed by Pyrkov et al. outperformed the architecture designed by Rahman and Adjeroh on the UKB dataset, which explains in part why we report higher prediction performances than Rahman and Adjeroh. Our better RMSE values than Pyrkov et al. (6.11±0.1 years vs. 14.0 years) despite using the same CNN architecture is likely driven by the narrower UKB’s age, as well as reasons (1) and (2) mentioned above (sample size and difference in time series types).

Gait changes as one gets older [5] and several research teams have predicted chronological age from the gait [10–18]. Lu and Tan analyzed the gait of 1,870 silhouette gaits collected from participants aged 20–30 years to predict chronological age with a MAE of 3.02 years [13]. Makihara et al. analyzed 1,728 gait videos collected from participants aged 2–94 years and predicted chronological age with a MAE of 8.2 years [16]. Riaz et al. attached integrated measurement units to the lower back of 26 participants (mean age: 48.1 years old; standard deviation: 12.7 years) and recorded their gait. They then trained a random forest on the features extracted from these records and classified the participants into three age categories (age < 40 years, 40 years < age < 50 years and age > 50 years) with 88.82% accuracy [17]. Hoffmann et al. recorded the gait of 142 participants aged 13–82 years as they walked over a sensor floor. They then built a neural network and predicted chronological age with a MAE of 9.55 years and a R^2^ value of 37.21% [19]. Li et al. built a support vector machine on features extracted from 63,846 gait energy image [18] recordings of participants aged 2–90 year and they predicted chronological age with a MAE of 6.78 years.

We analyzed the gait of the 77,269 UKB participants using wrist accelerometer recordings and predicted chronological age with a R^2^ of 32.3±0.2% and a MAE of 5.03±0.06 years. Only Hoffmann et al. reported a R^2^ value (37.21%) [19], larger than the one we obtained (32.3±0.2%), which can be explained by the wider age range covered by their dataset (13–82 years). The RMSE we obtained is lower than the MAE values reported by the aforementioned publications, which can be explained by the narrower age range covered by UKB (37–82 years).

Our study has a number of strengths and weaknesses. First, a notable strength is evaluation of multiple approaches of prediction of age using physical activity intensity and gait information across a large sample size of participants. The physical activity data is observed during a week-long observational period, which allows researchers to capture behavior during the week and weekend; however, individuals who are older may not be as active on the weekend vs. the week than their younger individuals. Second, we have attempted to find genetic and non-genetic correlates of our new measure of activity-based age, identifying genetic variants in a complex and variable region of the genome associated with olfactory, histone, and immune-related genes. Relatedly, the new measure of aging is a heritable phenotype. We note, however, that many of the correlations between non-genetic variables and the age prediction were modest and small. A weakness includes lack of representation of individuals under the age of 40–45 and consideration of how the age prediction may change as a function of chronological age itself. A major weakness that exists in the field and not limited to our own investigation includes assessing causality of the age prediction, a prerequisite for identifying drugs to reverse aging. If reversable, summarizing complex physical activity into a biological age predictor may be a way of observing the effect of preventative efforts in real-time, and, in the future, we aim to understand the clinical relevance of the age predictor by associating it with clinical outcomes.

## Supporting information

S1 Text Supporting information, including supporting Methods. (DOCX)

S1 Fig Age distribution of the UK Biobank cohort analyzed in this study in males (top) and females (bottom).
(DOCX)

S2 Fig Scatterplot of predicted physical activity-based age versus chronological age. R2: 63%; MAE: 3.73 years; RMSE: 4.71.
(DOCX)

S3 Fig Attention map samples for the images derived from the wrist accelerometer records. (DOCX)

S4 Fig Zoomed in “LocusZoom” Manhattan Plot of loci implicated on chromosome 6. Genes locations are also depicted on the bottom of the plot. Color of point denotes linage disequilibrium, or correlation between loci identified in GWAS.
(PDF)

S5 Fig Attention map example for physical activity time series—Full week. The participant is a correctly predicted 60-65-year-old male. Red data points represent time steps for which a higher value would increase the chronological age prediction, and blue data points represent time steps for which a higher value would decrease the chronological age prediction.
(DOCX)

S6 Fig Correlation between the different definitions of accelerated aging. (DOCX)

S7 Fig Sample preprocessed one-dimensional acceleration time series generated from a participant’s wrist accelerometer recording. The participant is a 65-70-year-old male.
(DOCX)

S8 Fig Sample preprocessed three-dimensional acceleration time series generated from a participant’s wrist accelerometer recording during their walk. The participant is a 40-45-year-old female participant.
(DOCX)

S9 Fig Recurrence plot, Markov transition field and Gramian angular field (difference and summation) generated from a participant’s wrist accelerometer recording. The participant is a 65-70-year-old male. For the recurrence plot, the upper right triangle corresponds to the sleeping timesteps, and the lower left triangle corresponds to the remaining time steps.
(DOCX)

S10 Fig Architecture of the convolutional neural network trained on the raw wrist accelerometer acceleration time series—Summary figure. (DOCX)

S11 Fig Architecture of the convolutional neural network trained on the raw wrist accelerometer acceleration time series—Detailed figure. (PDF)

S12 Fig Architecture of the convolutional neural network trained on the wrist accelerometer features time series—Summary figure. (DOCX)

S13 Fig Architecture of the convolutional neural network trained on the wrist accelerometer features time series—Detailed figure. (PDF)

S14 Fig Architecture of the convolutional neural network trained on the wrist accelerometer 3D raw acceleration time series—Summary figure. (DOCX)

S15 Fig Architecture of the convolutional neural network trained on the wrist accelerometer 3D raw acceleration time series—Detailed figure. (PDF)

S16 Fig QQplot of association from GWAS. Lambda genomic control was 1.04. (DOCX)

S17 Fig Lambda GC versus A) INFO and B) Minor Allele Frequency.
(DOCX)

S1 Table R-squared (R2), root-mean squared error (RMSE), and mean absolute error (MAE) for each model interrogated. Ensemble models are denoted by *. Estimates plus/minus standard deviation of the estimate across the folds.
(XLS)

S2 Table Scalar features generated from the wrist accelerometer data. (XLSX)

S3 Table Feature importances for the models built on scalar predictors. See supplementary data. (XLSX)

S4 Table GWAS SNPs by chromosome and position. Chr: denotes chromosome number; Position: coordinate on chromosome, EA;NEA: Effect Allele;Non-effect Allele; MAF: Minor Allele Frequency; Beta: Beta coefficient of association; SE: Standard error of Beta; Pvalue: Pvalue of association, nSNPs: number of SNPs in linkage. (XLSX)

S5 Table List of biomarkers by subcategories for the Biomarkers Wide Association Study. (XLSX)

S6 Table Biomarkers associated with accelerated aging. (XLSX)

S7 Table Biomarkers associated with decelerated aging. (XLSX)

S8 Table List of clinical phenotypes by subcategories for the Clinical Phenotypes Wide Association Study. (XLSX)

S9 Table Clinical phenotypes associated with accelerated aging. (XLSX)

S10 Table Clinical phenotypes associated with decelerated aging. (XLSX)

S11 Table List of diseases by subcategories for the Diseases Wide Association Study. (XLSX)

S12 Table Diseases associated with accelerated aging. (XLSX)

S13 Table List of family history variables by subcategories for the Family History Phenotypes Wide Association Study. (XLSX)

S14 Table List of environmental variables by subcategories for the Environmental Wide Association Study. (XLSX)

S15 Table Environmental variables associated with accelerated aging. (XLSX)

S16 Table Environmental variables associated with decelerated aging. (XLSX)

S17 Table List of socioeconomic variables by subcategories for the Socioeconomics Wide Association Study. (XLSX)

S18 Table Socioeconomic variables associated with accelerated aging. (XLSX)

S19 Table Socioeconomic variables associated with decelerated aging. (XLSX)

S20 Table Exhaustive XWAS results—association between non-genetic factors and accelerated aging. (XLSX)

S21 Table Physical activity scalar features outputted by UKB’s software and removed from our list of predictors because non-biological. (XLSX)

S22 Table Comparison between our age predictors and the literature in terms of prediction performance. (XLSX)

S23 Table Hyperparameter space for scalar features-based models Bayesian optimization. (XLSX)

S24 Table Nested Cross-Validation pipeline. (XLSX)

S25 Table Hyperparameters tuning upstream of the cross-validation for images-based models. (XLSX)

S26 Table Outer Cross-Validation with inner split pipeline. The values displayed are validation RMSE values. Lower values are associated with better hyperparameter tuning. When two values are displayed (value1/value2), the second value corresponds to the training RMSE. The architecture used was InceptionV3, with an initial learning rate of 0.001. The model was trained on the data folds 2–9, and validated on the data fold #0. The data fold #1 was set aside as the testing set and was not used.
(XLSX)
