# Supplementary material for: Machine learning approaches to predict age from accelerometer records of physical activity at biobank scale
Source: PLOS Digit Health. 2023 Jan 24;2(1):e0000176. doi: 10.1371/journal.pdig.0000176 (PMC9931315; doi:10.1371/journal.pdig.0000176)
Supplement: S1 Fig — Age distribution of the UK Biobank cohort analyzed in this study in males (top) and females (bottom). (DOCX) [file pdig.0000176.s002.docx]

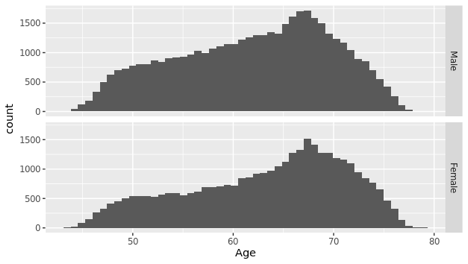


S1 Figure. Age distribution of the UK Biobank cohort analyzed in this study in males (top) and females (bottom).
