# Supplementary material for: Machine learning approaches to predict age from accelerometer records of physical activity at biobank scale
Source: PLOS Digit Health. 2023 Jan 24;2(1):e0000176. doi: 10.1371/journal.pdig.0000176 (PMC9931315; doi:10.1371/journal.pdig.0000176)
Supplement: S2 Fig — R2: 63%; MAE: 3.73 years; RMSE: 4.71. (DOCX) [file pdig.0000176.s003.docx]

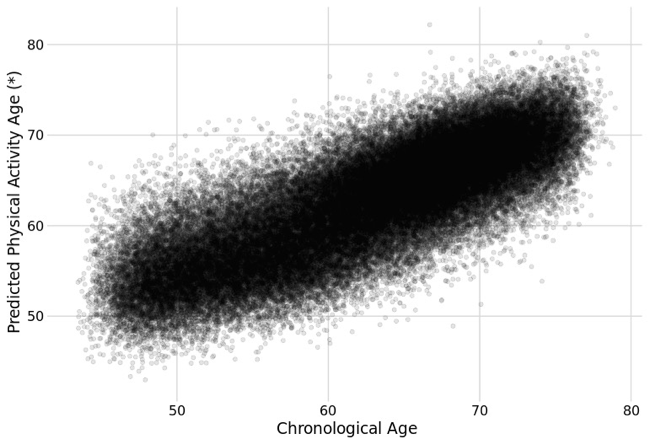


S2 Figure. Scatterplot of predicted physical activity-based age versus chronological age. R^2^: 63%; MAE: 3.73 years; RMSE: 4.71
