# Supplementary material for: Machine learning approaches to predict age from accelerometer records of physical activity at biobank scale
Source: PLOS Digit Health. 2023 Jan 24;2(1):e0000176. doi: 10.1371/journal.pdig.0000176 (PMC9931315; doi:10.1371/journal.pdig.0000176)
Supplement: S3 Fig — (DOCX) [file pdig.0000176.s004.docx]

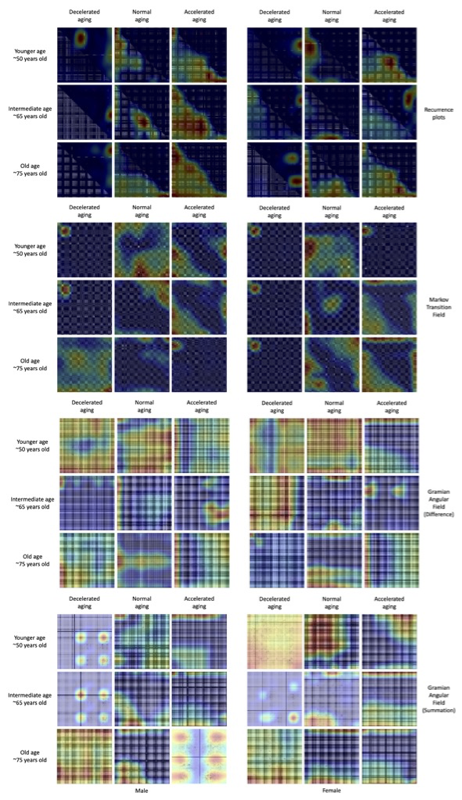


[S3](#sfigu_attention_maps) Figure**:** Attention map samples for the images derived from the wrist accelerometer records
