# Supplementary figures and images for: Machine learning approaches to predict age from accelerometer records of physical activity at biobank scale
Source: PLOS Digit Health. 2023 Jan 24;2(1):e0000176. doi: 10.1371/journal.pdig.0000176 (PMC9931315; doi:10.1371/journal.pdig.0000176)

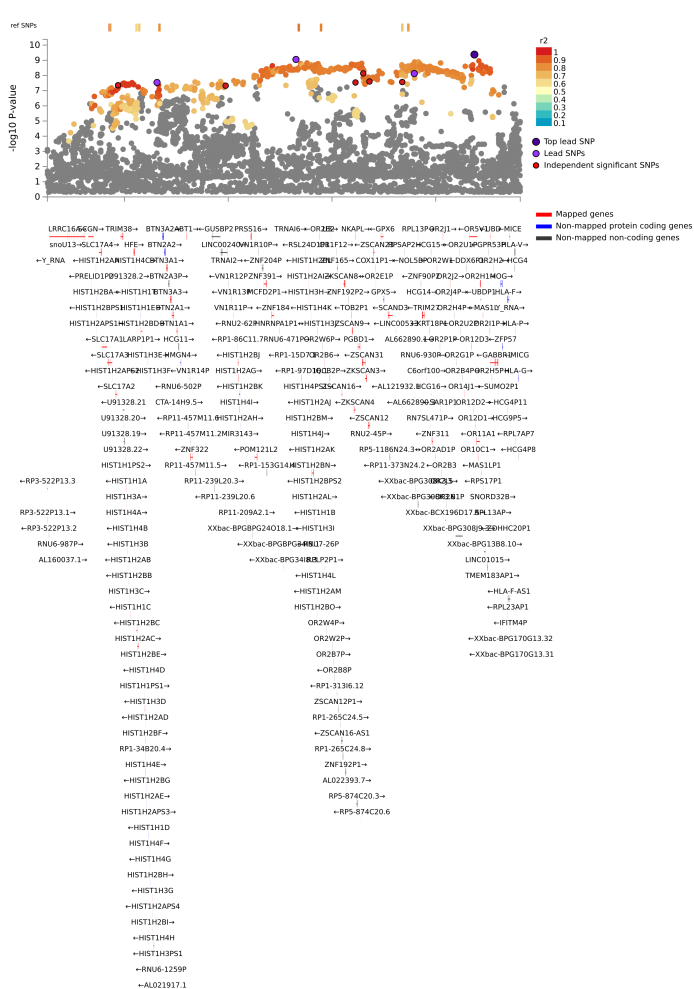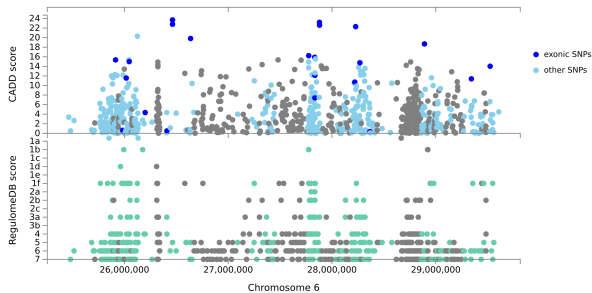

Supplement: S4 Fig — Genes locations are also depicted on the bottom of the plot. Color of point denotes linage disequilibrium, or correlation between loci identified in GWAS. (PDF) [file pdig.0000176.s005.pdf]

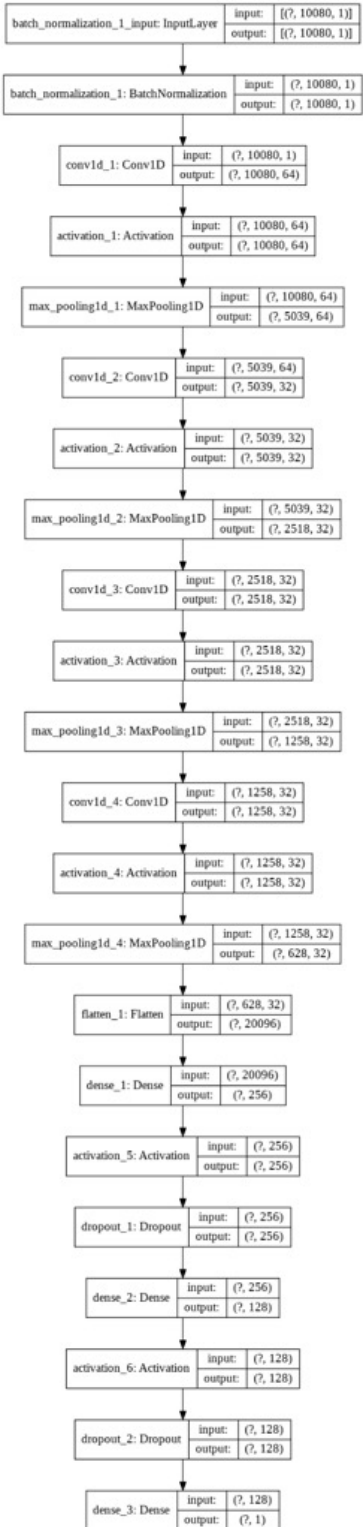

Supplement: S11 Fig — (PDF) [file pdig.0000176.s012.pdf]

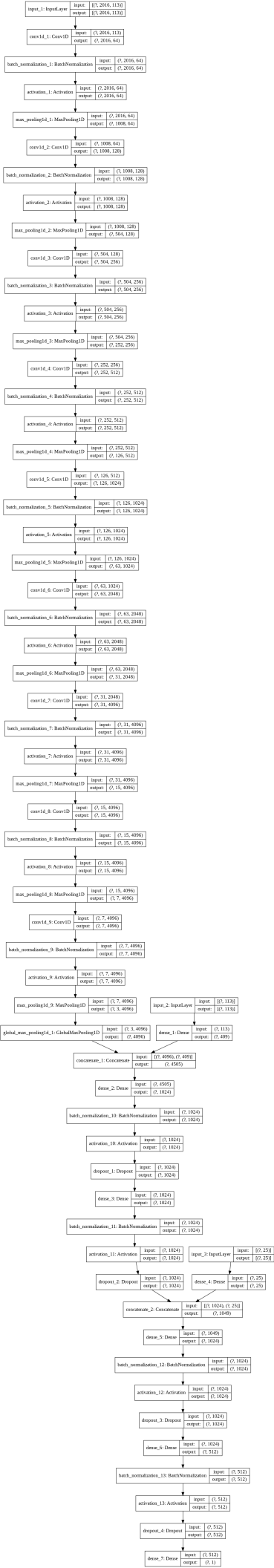

Supplement: S13 Fig — (PDF) [file pdig.0000176.s014.pdf]

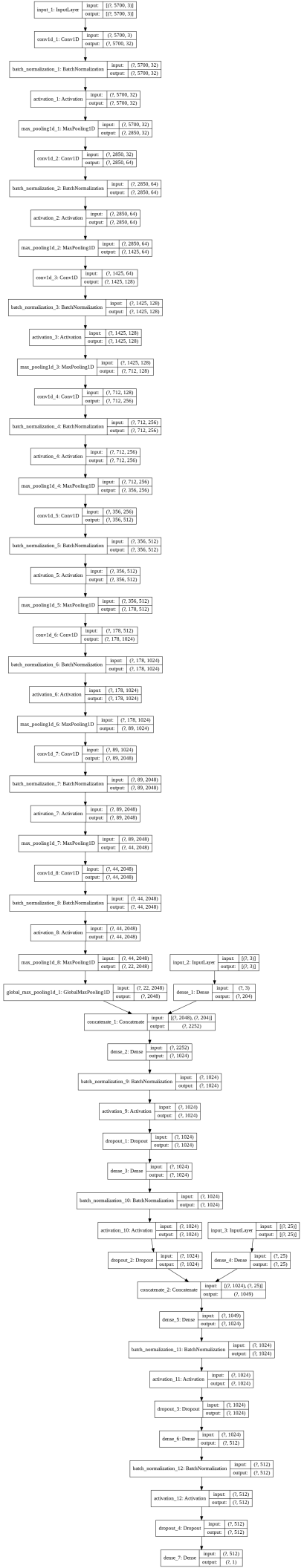

Supplement: S15 Fig — (PDF) [file pdig.0000176.s016.pdf]

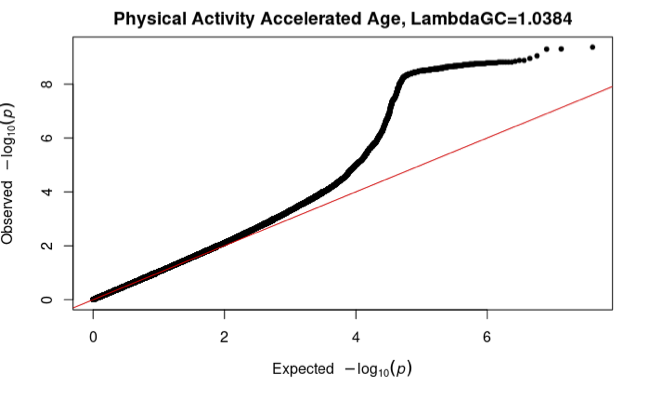


[S16](#sfigu_gwas_qqplot) Figure: QQplot of association from GWAS. Lambda genomic control was 1.04.

Supplement: S16 Fig — (DOCX) [file pdig.0000176.s017.docx]

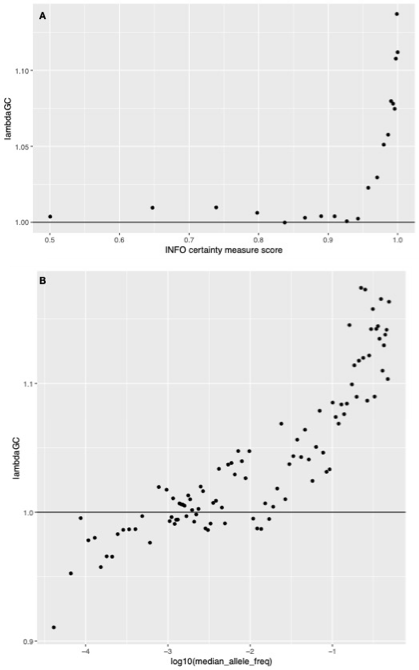


[S17](#sfigu_gwas_qc) Figure. Lambda GC versus A) INFO and B) Minor Allele Frequency.

Supplement: S17 Fig — Lambda GC versus A) INFO and B) Minor Allele Frequency. (DOCX) [file pdig.0000176.s018.docx]
