# Supplementary material for: Machine learning approaches to predict age from accelerometer records of physical activity at biobank scale
Source: PLOS Digit Health. 2023 Jan 24;2(1):e0000176. doi: 10.1371/journal.pdig.0000176 (PMC9931315; doi:10.1371/journal.pdig.0000176)
Supplement: S5 Fig — Red data points represent time steps for which a higher value would increase the chronological age prediction, and blue data points represent time steps for which a higher value would decrease the chronological age prediction. (DOCX) [file pdig.0000176.s006.docx]

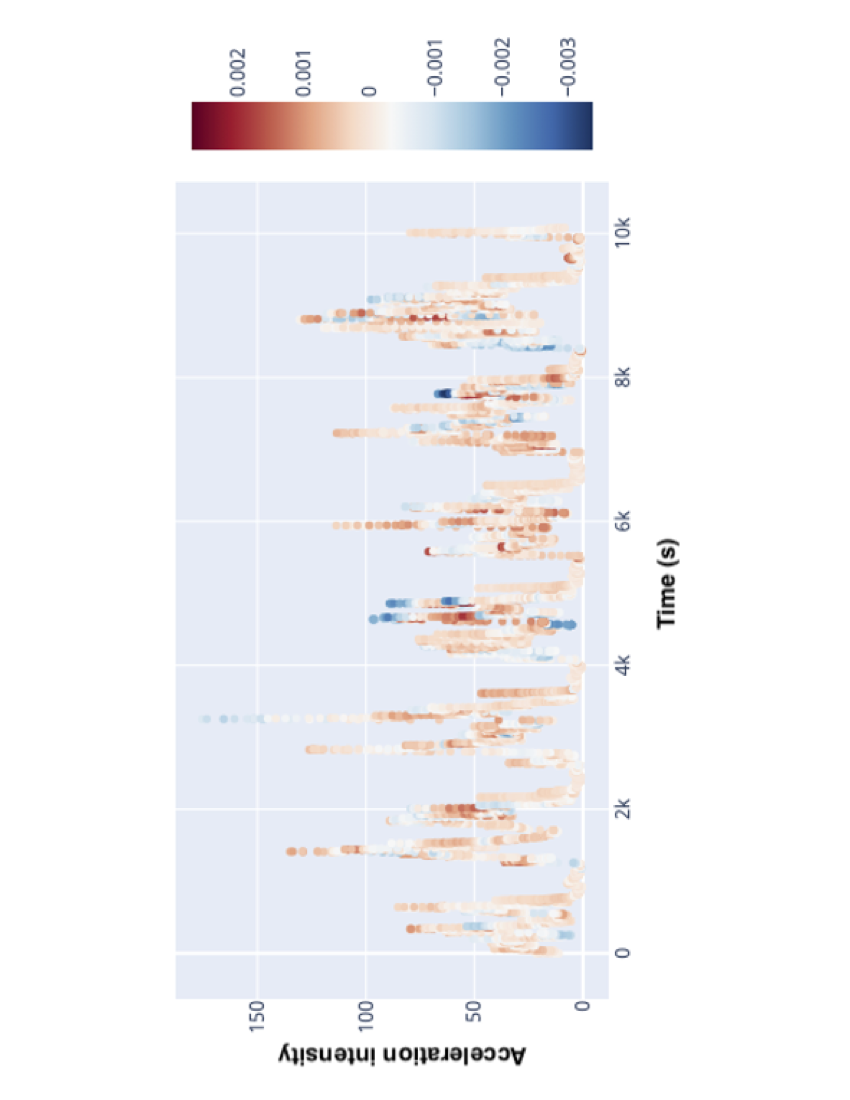


[S5](#sfigu_attentionmaps_physicalactivity_fullweek) Figure**:** Attention map example for physical activity time series - Full week. The participant is a correctly predicted 60-65-year-old male. Red data points represent time steps for which a higher value would increase the chronological age prediction, and blue data points represent time steps for which a higher value would decrease the chronological age prediction.
