# Supplementary material for: Machine learning approaches to predict age from accelerometer records of physical activity at biobank scale
Source: PLOS Digit Health. 2023 Jan 24;2(1):e0000176. doi: 10.1371/journal.pdig.0000176 (PMC9931315; doi:10.1371/journal.pdig.0000176)
Supplement: S7 Fig — The participant is a 65-70-year-old male. (DOCX) [file pdig.0000176.s008.docx]

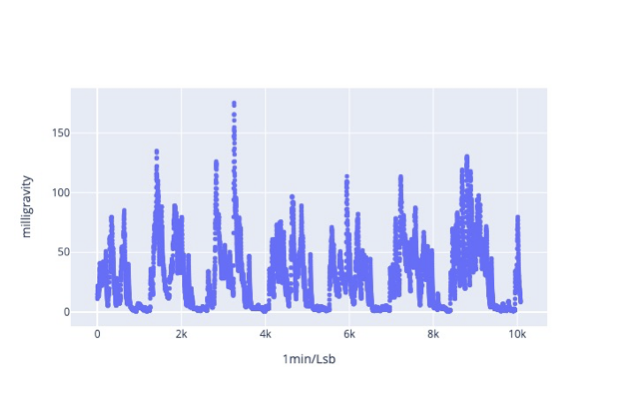


[S7](#sfigu_PA_1Dacceleration) Figure: Sample preprocessed one-dimensional acceleration time series generated from a participant’s wrist accelerometer recording. The participant is a 65-70-year-old male.
