# Supplementary material for: Machine learning approaches to predict age from accelerometer records of physical activity at biobank scale
Source: PLOS Digit Health. 2023 Jan 24;2(1):e0000176. doi: 10.1371/journal.pdig.0000176 (PMC9931315; doi:10.1371/journal.pdig.0000176)
Supplement: S8 Fig — The participant is a 40-45-year-old female participant. (DOCX) [file pdig.0000176.s009.docx]

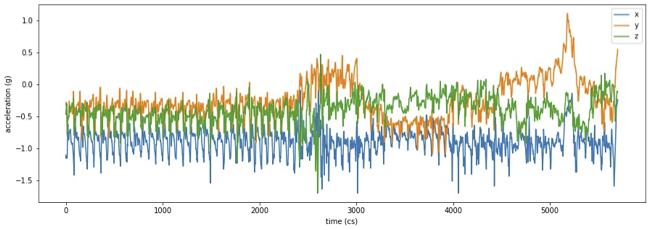


[S8](#sfigu_PA_3Dacceleration) Figure: Sample preprocessed three-dimensional acceleration time series generated from a participant’s wrist accelerometer recording during their walk

The participant is a 40-45-year-old female participant.
