# Supplementary material for: Machine learning approaches to predict age from accelerometer records of physical activity at biobank scale
Source: PLOS Digit Health. 2023 Jan 24;2(1):e0000176. doi: 10.1371/journal.pdig.0000176 (PMC9931315; doi:10.1371/journal.pdig.0000176)
Supplement: S9 Fig — The participant is a 65-70-year-old male. For the recurrence plot, the upper right triangle corresponds to the sleeping timesteps, and the lower left triangle corresponds to the remaining time steps. (DOCX) [file pdig.0000176.s010.docx]

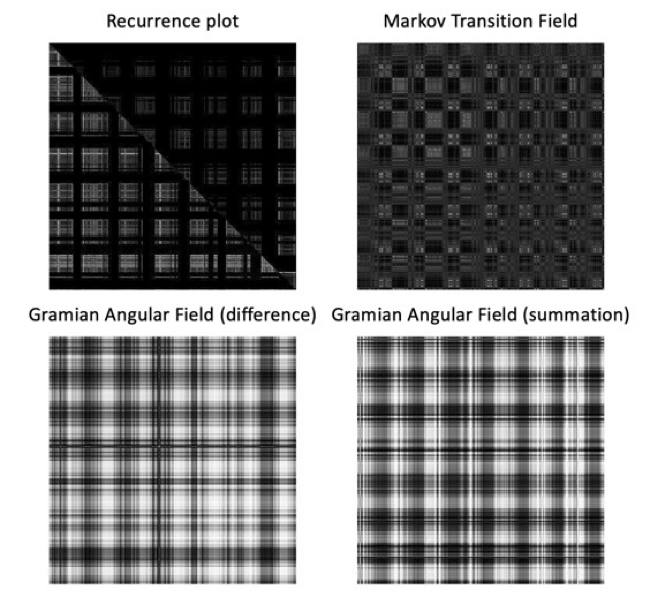


[S9](#sfigu_PA_images) Figure**:** Recurrence plot, Markov transition field and Gramian angular field (difference and summation) generated from a participant’s wrist accelerometer recording

The participant is a 65-70-year-old male. For the recurrence plot, the upper right triangle corresponds to the sleeping timesteps, and the lower left triangle corresponds to the remaining time steps.
