# Supplementary material for: Machine learning approaches to predict age from accelerometer records of physical activity at biobank scale
Source: PLOS Digit Health. 2023 Jan 24;2(1):e0000176. doi: 10.1371/journal.pdig.0000176 (PMC9931315; doi:10.1371/journal.pdig.0000176)
Supplement: S12 Fig — (DOCX) [file pdig.0000176.s013.docx]

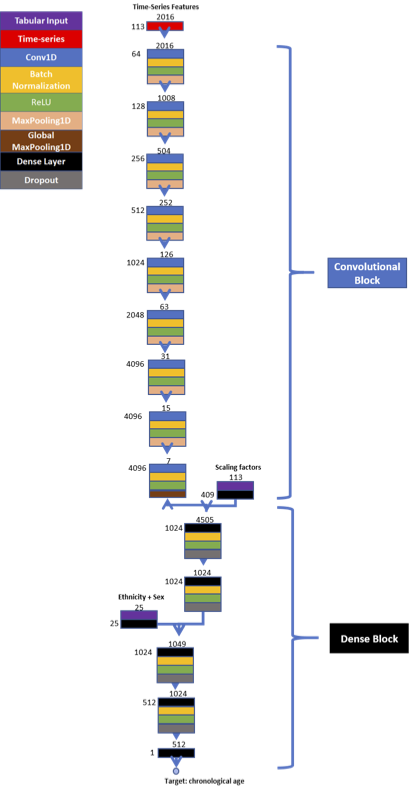


[S12](#sfigu_architecture_PAfullweek_features_summary) Figure: Architecture of the convolutional neural network trained on the wrist accelerometer features time series - Summary figure
